# Antibiotic Resistance Genetic Markers and Integrons in White Soft Cheese: Aspects of Clinical Resistome and Potentiality of Horizontal Gene Transfer

**DOI:** 10.3390/genes9020106

**Published:** 2018-02-19

**Authors:** Ana Caroline L. de Paula, Julliane D. Medeiros, Analice C. de Azevedo, Jéssica M. de Assis Chagas, Vânia L. da Silva, Cláudio G. Diniz

**Affiliations:** Department of Parasitology, Microbiology and Immunology, Institute of Biological Sciences, Federal University of Juiz de Fora, 36036-900 Juiz de Fora, MG, Brazil; carol.lopesdp@gmail.com (A.C.L.d.P.); jdutramedeiros@gmail.com (J.D.M.); analiceazevedo@hotmail.com (A.C.d.A.); jessica.chagas@hotmail.com.br (J.M.d.A.C.); vania.silva@ufjf.edu.br (V.L.d.S.)

**Keywords:** antibiotic resistance, genetic markers, Minas Frescal Cheese, integron

## Abstract

Antibiotic resistance poses an important threat to global public health and has become a challenge to modern medicine. The occurrence of antibiotic-resistant bacteria in a broad range of foods has led to a growing concern about the impact that food may have as a reservoir of antibiotic resistance genes. Considering Minas Frescal Cheese (MFC)—a typical Brazilian white soft cheese—and its economic and cultural values, in this study, medically relevant antimicrobial-resistance genetic markers (AR genes) were screened, and the occurrence of integrons were evaluated in manufactured MFC using culture-independent approaches. Through a fingerprinting analysis, the tested MFCs were brand-clustered, indicating reproducibility along the production chain. A common core of resistance markers in all brands evaluated and related antimicrobials such as β-lactams, tetracyclines, quinolones, and sulfonamide was detected. Several other markers, including efflux pumps and aminoglycosides-resistance were distributed among brands. Class 1 and 2 integrons were observed, respectively, in 77% and 97% of the samples. The presence of AR genes is of special interest due to their clinical relevance. Taken together, the data may suggest that the production chain of MFC might contribute to the spread of putative drug-resistant bacteria, which could greatly impact human health. Furthermore, detection of class 1 and class 2 integrons in MFC has led to discussions about resistance gene spread in this traditional cheese, providing evidence of potential horizontal transfer of AR genes to human gut microbiota.

## 1. Introduction

As long as antimicrobial-resistant bacteria are being detected worldwide, associated to activities other than health-care and including food chain production, the phenomena will continue to pose a major threat to humans, animals, and environmental health and will become the focus of scientific investigation [1]. For several decades, studies on the dissemination of antibiotic resistance have focused mainly on health-care facilities and clinically relevant bacterial species [2]. However, it has been well documented that antimicrobials widely used in livestock have contributed to the spread of antibiotic resistant bacteria or antimicrobial resistance genes throughout the food production chain up to consumers and surrounding environments. In addition, it is accepted that foodstuff may play an important role in the phenomena since ready to eat food may be cross contaminated with antimicrobial resistant bacteria during processing, becoming an important reservoir and raising questions about its safety [2,3,4,5]. 

Antimicrobial resistance in foodborne pathogens involves direct risk for consumers’ health, impairs successful antibiotic treatment of infections, and increases the number of hospitalizations [6]. In addition, commensal bacteria found in the food may act as reservoirs for resistant genes which may potentially be transferred to gut microbiota and putative pathogens through horizontal gene transfer [7,8,9]. 

Resistance-encoding genes can be located on chromosomes or on mobile genetic elements, such as plasmids, transposons, and integrons, making them available for horizontal gene transfer [10]. Integrons are genetic platforms that may integrate gene cassettes (which are promoterless genetic elements) mainly related to antimicrobial resistance; they contain promoter(s) allowing the expression of those inserted cassettes and may be spread and incorporated into transposons and plasmids [11,12].

Dairy products such as milk and cheese have been implicated as potential sources for transmission of antimicrobial-resistant bacteria, with a direct link between the animal microbiota and the human gastrointestinal tract [13]. Minas Frescal Cheese (MFC), a white soft fresh cheese, is one of the most consumed and produced cheeses in Brazil. Due to the low salt content and high moisture, the MFC is an appropriate environment for the growth of putative pathogens. In addition, their manufacture involves technological steps that require the use of equipment without efficient treatments to reduce the microbial load [14,15,16]. Although sanitary legislations consider only classical foodborne pathogens such as coagulase-positive *Staphylococcus*, *Listeria monocytogenes*, and coliforms as quality indicators, other drug-resistant putative bacteria (DRB) may occur, posing a health issue [17,18,19]. 

To date, despite MFC having already been associated with multidrug-resistant bacteria, and considering the major microbial groups related to human health, there are no data on the cheese resistome, or even the clinical resistome including the occurrence of integrons, which would potentially be associated with antimicrobial-resistance genetic marker (AR genes) transfer. Most of the studies evaluating the prevalence of AR genes in cheese samples are based on methodological approaches such as isolation and identification of the resistant bacteria, followed by a molecular analysis of their AR determinants [18,20,21,22,23]. In this regard, a holistic view of cheese-resistome based on culture-independent approaches would retrieve valuable information on its quality, as those drug-resistant associated genes may be harbored by classical pathogens and by putative bacteria (not considered in food sanitary legislations) [20,24]. 

To contribute to aspects of clinical resistome and microbiological safety of manufactured MFC, this study aimed to perform a screening of clinically relevant antimicrobial-resistance genetic markers and evaluate the occurrence of integrons by culture-independent techniques based on direct analysis of total DNA from cheese samples. In addition, the application of the repetitive extragenic palindromic sequences polymerase chain reaction (rep-PCR) fingerprinting technique to compare the microbial community profile of MFC samples and evaluate its reproducibility was also evaluated.

## 2. Materials and Methods

### 2.1. Sample Processing and DNA Extraction

Samples from 35 cheeses from seven different commercially manufactured, trademarked, Minas soft cheeses (A, B, C, D, E, F, and G) were purchased in retail stores in Juiz de Fora (Minas Gerais state, Brazil). To obtain Brazilian sanitary certification, cheeses are made from pasteurized bovine milk. Following acquisition, the samples were kept in an ice box and sent to the laboratory for immediate processing. Total DNA was extracted directly from 5 samples (same batch) of seven different cheese brands. Twenty-five-gram cheese portions were weighted and homogenized in 225 mL of 2% (*w*/*v*) sterile sodium citrate (pH 7,5), using a shaker at 250 rpm for 20 min. Aliquots (50 mL) were centrifuged (14,000× *g*, 10 min), and the supernatant containing the fat layer was discarded. The pellet obtained was resuspended in 10 mL of Tris-EDTA (TE) (10 mM Tris; 1 mM ethylenediaminetetraacetic acid (EDTA); pH 8) and then centrifuged at 14,000× *g* for 10 min. This step (addition of wash TE buffer and centrifugation) was repeated twice, and the obtained pellet was resuspended in 200 µL TE buffer. DNA was extracted from the cell pellet using the QIAamp DNA Stool Mini Kit (Qiagen, Hilden, Germany) according to the protocol. DNA concentration was determined by fluorimetry using the QubitTM 2.0 fluorimeter, with the Qubit dsDNA HS Assay kit (Life Technologies, Carlsbad, CA, USA). DNA integrity was assessed by 0.8% agarose gel electrophoresis. The resulting purified DNA was stored at −70 °C until use. 

### 2.2. Repetitive Extragenic Palindromic Sequences Polymerase Chain Reaction

The rep-PCR analysis was performed using the total DNA extracted directly from the cheese samples. The PCR reactions were performed according to Gaber et al. [25], with modifications, using a single primer (GTG)5 (5′-GTGGTGGTGGTGGTG-3′). PCR final concentration contained 1× Master Mix (Promega, Madison, WI, USA), 0.8 μM of the primer, 20 ng of DNA, and ultrapure PCR water (Promega) was added to a final volume of 25 μL. The PCR conditions were: 5 min at 94 °C; 30 cycles of 1 min at 94 °C; 1 min at 50 °C; 2.5 min at 72 °C; and a final extension of 10 min at 72 °C. The PCR products were checked by electrophoresis in 2% (*w*/*v*) agarose gel for 2 h at a constant voltage of 100 V in 1× Tris/Acetate/EDTA buffer (TAE). A 100 bp DNA ladder (Ludwig Biotec, Porto Alegre, RS, Brazil) was used as a molecular-size marker. Gels were stained using ethidium bromide, and the images were visualized in ultraviolet light transilluminator (GE Healthcare, Little Chalfont, Bucks, UK). Fingerprints were analyzed using BioNumerics 5.1 (Applied Maths, Sint-Martens-Latem, Belgium). The similarities between the profiles were calculated using the Jaccard coefficient, and the dendrograms were constructed using the Unweighted Pair Group Method with Arithmetic Mean (UPGMA).

### 2.3. Screening of Antibiotic Resistance Genetic Markers and Integrons

A set of 40 resistance genetic markers of human clinical relevance (clinical resistome), including genetic markers related to efflux pumps and class 1, 2, and 3 integrons, were screened from the metagenomic DNA of the samples by conventional PCR, using specific primers and amplification conditions previously described (Supplementary Table S1). PCR products were analyzed by agarose gel electrophoresis 1.5% (*w*/*v*) in 1× Tris/Borate/EDTA (TBE) buffer. DNA bands were visualized after gel staining with ethidium bromide on ultraviolet light transilluminator. Amplicon sizes were estimated by a 100 bp DNA ladder (Ludwig Biotec). Negative controls were performed as reactions without template DNA. As a positive control, amplicons were purified using the QIAquick Gel Extraction Kit (Qiagen), and the DNA fragments obtained were sequenced in an ABI Prism 3730 DNA sequencer (Applied Biosystems, Foster City, CA, USA) and were further blasted against the nucleotide database of the National Center for Biotechnology Information (NCBI). The clustering based on the occurrence of antibiotic resistance markers was performed by a principal component analysis (PCA) using a PAST program (version 3.14).

## 3. Results

**Figure 1:**
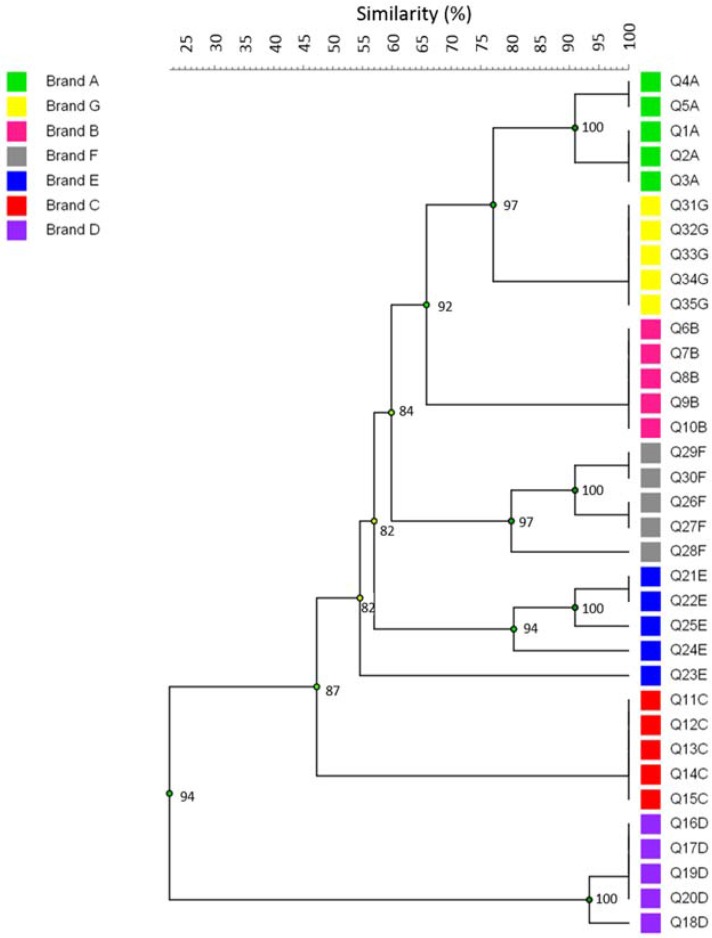
Clustering of microbial community profile from different brands of Minas Frescal Cheese (MFC). The dendrogram was obtained from repetitive extragenic palindromic sequences polymerase chain reaction (rep-PCR) DNA fingerprint data, using the Unweighted Pair Group Method with Arithmetic Mean (UPGMA) grouping method.

Repetitive extragenic palindromic sequences PCR was considered in this study as a culture-independent method, using the total DNA extracted from the cheese to compare the microbial community profile among samples from the same batch and to provide evidence of its reproducibility. The dendrogram obtained by rep-PCR clustering of cheese samples is presented in Figure 1. In general, considering a coefficient of similarity greater than 80%, the samples were clearly brand-clustered, suggesting standardization along the production chain and that the sampling in this study was reproducible. This result is in agreement with that expected since the products from the same batch are processed under similar conditions. However, one sample from brand E differed from the others, clustering with 55% similarity.

**Figure 2:**
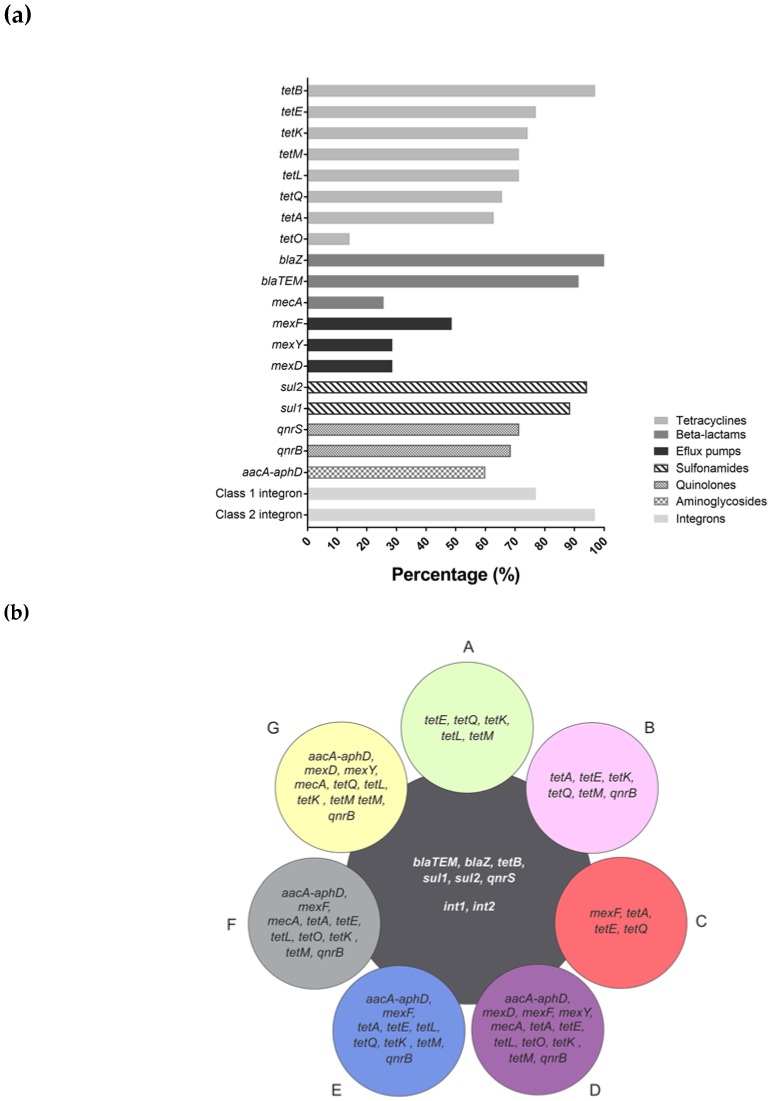
Clinical resistome composition of MFC. (**a**) Frequency of antimicrobial-resistance genetic markers detection based on their pharmacological properties; (**b**) Occurrence profile of antimicrobial-resistance markers among the cheese brands (A to G).

Considering the occurrence of antibiotic resistance markers, Figure 2a shows the percentage of resistance markers obtained in the 35 samples analyzed, according to the pharmacological class. From 40 resistance markers investigated, 14 were detected related to tetracyclines, β-lactams, efflux pumps, sulfonamides, quinolones, and aminoglycosides. On the other hand, resistance markers to macrolide, lincosamide, streptogramin, and erythromycin were not detected in any sample. In particular, the *blaZ* β-lactamase gene was detected in 100% of the samples and *tetB, blaTEM, sul1*, and *sul2* genes in more than 80%. Despite the high percentage of occurrence, only three resistance markers related to β-lactams were detected, representing 21.4% of the total markers evaluated for this class (14 markers). Resistance markers to tetracyclines were the most prevalent in the analyzed samples. All markers evaluated in this study (eight markers) were detected among the samples, despite the low occurrence of the *tetO* gene.

**Figure 3:**
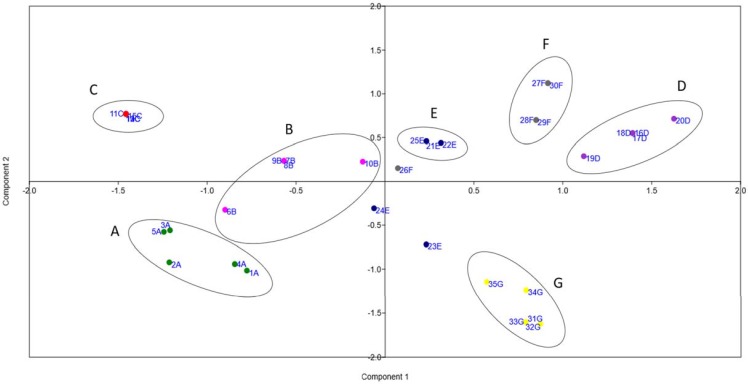
Clustering of cheese samples according to the occurrence of antibiotic resistance markers using principal component analysis (PCA). Green: brand A (1A to 5A); pink: brand B (6B to 10B); red: brand C (11C to 15C); purple: brand D (16D to 20D); blue: brand E (21E to 25E); grey: brand F (26F to 30F); yellow: brand G (31G to 35G).

The co-occurrence of different resistance genes was observed in the analyzed brands and each one presented a particular profile (Figure 2b). In general, the frequency of detection and the variety of markers were higher in the brand D samples and lowest in brand C. Nevertheless, a common core of antibiotic resistance genes, related to tetracycline, β-lactam, quinolone, and sulfonamide (*tetB, bla-TEM, blaZ, qnrS, sul1,* and *sul2*), were detectable in all brands (Figure 2b). In accordance with the clustering obtained by the rep-PCR fingerprint, the samples were also brand-clustered according to the occurrence profile of resistance markers, as observed in Figure 3.

PCR-based detection of integrons was carried out on all the samples, using specific primers for three different *intI* genes (*intl1, intl2 and intl3*). Class 1 and 2 integrons were detected, respectively, in 77% and 97% of the samples and also characterized the common core of detectable genes in all brands (Figure 2b). Class 3 integrons were not detected in any sample analyzed.

## 4. Discussion

In general, microbiota characterization, especially in food microbiology, using culture-dependent approaches may not reveal the overall microbial diversity due to its complexity. On the other hand, culture-independent methods based on molecular biology techniques, such as rep-PCR, have been suggested to globally characterize the microbial population and differentiate cheese samples [26,27]. 

In this study, a rep-PCR approach as a culture-independent method to compare the microbial community profile of MFC samples and evaluate its reproducibility in the same batches was employed. Of the tested samples, only one from brand E did not follow the 80% similarity criteria within the same batch. This sample clustered with a similarity of 55%, which may represent low quality control during manufacturing, addressing issues related to microbial contamination along food chain production. However, a limitation in this approach was the lack of the assessment of which groups of microorganisms are present or absent in each sample.

Antibiotic resistance genes are widely distributed in several different environments, including food production systems. Food microbiology has also taken advantage of culture-independent, DNA-based molecular methods for the detection and analysis of AR genes directly in food samples, including several cheese types. Devirgiliis et al. [28] investigated the presence of specific antibiotic resistance genes in total DNA extracted from the whole microbiome present in an Italian cheese using molecular approaches. PCR detection of tetracycline and erythromycin resistance genes was performed in DNA from commercial Spanish and Italian cheeses samples by Flórez et al. [20]. In this study, a specific white soft cheese as a model, that is, MFC, which is typically produced in Brazil and consumed fresh was chosen.

Several genetic markers related to antimicrobial resistance have been detected, namely, the *tet* genes, which are associated with ribosomal protection and tetracycline efflux mechanisms. These were the most prevalent among the processed MFC. Although tetracyclines are well-known for their broad spectrum of activity to Gram-positive and Gram-negative bacteria, the spread of tetracycline-resistant mechanisms limited their use in treating bacterial infections [29]. Worldwide use of tetracycline in livestock production is well-known and leads to the persistence of resistance in the food chain and facilitates its spread among human pathogens. The tetracycline resistance genes have been reported in lactic acid bacteria (LAB) strains isolated from dairy products [28,30] including artisanal and industrial cheeses [20,31].

β-lactams are also clinically relevant antibiotics, for which resistance genes have also been described in bacteria from several foods, including cheeses. The *mecA* gene related to methicillin resistance, detected in 25.7% of the cheese samples in this study, was previously associated with coagulase-negative *Staphylococci* in this kind of cheese [18]. Add to that, the *blaTEM*, related to extended spectrum β-lactamases (ESBL), was detected in 91.4% of the samples. In general, ESBL is mainly encoded by mobile genetic elements such as integrons, transposons, and plasmids, which are easily transferable to other bacteria. Occurrence of *blaTEM* had already been documented in bacteria isolated from Portuguese soft and semi-soft cheeses [21]. Of especial interest, another β-lactams resistance gene, *blaZ,* was observed in all tested cheese sample. The data is impressive and may be related to the massive use of penicillin in Brazil, both in animal and human medicine. In cheese samples, *blaZ* has been reported in isolates of *Staphylococcus aureus* [32,33,34].

The presence of the aminoglycoside resistance gene (*aacA-aphD*) in 60% of the cheese samples is also of concern due to its controlled use, although this gene was already associated with coagulase-negative *Staphylococci* isolated from cheese samples in Turkey [23]. Aminoglycosides are important drugs in human therapy and their use in animal husbandry has been strictly regulated to avoid the selection and spread of aminoglycoside-resistant bacteria [35].

Quinolone resistance genes, *qnrB* and *qnrS,* detected at rates of 68.6% and 71.4%, respectively, along with sulfonamide resistance genes, *sul1* and *sul2,* detected at rates of 88.6% and 94.3%, respectively, are also of particular interest; to date, these genetic markers have not been related to cheese. According to the literature, both *qnr* and *sul* genes are thought to be integron- and plasmid-born genes and, thus, highly available to horizontal transfer and environmental persistence upon selective pressure [36,37,38]. 

Bacterial multidrug efflux pumps are antibiotic resistance determinants which may be found in all microorganisms. These transporters can actively extrude a variety of substrates such as antibiotics, heavy metals, dyes, detergents, organic solvents, among others, indicating their importance in bacterial physiology under environmental selective pressure as a defense mechanism. Unspecific efflux pumps that transport several compounds can be associated with bacterial cross-resistance, i.e., simultaneous resistance to different antimicrobial drugs and other xenobiotics [39,40]. Efflux pump genes detected in this study encode Mex-type multidrug resistance efflux pumps (mexD, mexF, and mexY). These Mex systems can export several drugs such as chloramphenicol, fluoroquinolones, and tetracycline and are often described in opportunistic pathogenic bacteria [41,42].

Overall, the screening of AR genes in this study provided information on particularities of MFC processing. Although there is no available information with which to compare the results, taken together, the data may suggest that different manufacturing plants may face different contamination or even cross-contamination failures, leading to a peculiar AR profile in the final product which is available to the consumers. To date, it is not possible to address if the microbial contamination is related to the raw-material used in the dairy industry or related to human handling. However, the MFC resistome would be predictive of regional antimicrobial resistance epidemiology. 

In Brazil, despite the lack of statistical information about the use of antibiotics in livestock, the β-lactams and tetracyclines are drugs frequently chosen for the treatment of infections affecting dairy herds [43]. In this study, several resistance-markers related to both treatment and prophylaxis in human and veterinary medicine were detected. According to the literature, food chain contamination including antimicrobial-resistant bacteria may be related to: (i) the use of xenobiotics in dairy, livestock, and agriculture, or (ii) contamination in plant processing or handling [5].

It is well-known that genes encoding antimicrobial resistance are often linked to mobile genetic elements. In this study, class 1 and 2 integrons were detected in 77% and 97% of the cheese samples, respectively, raising issues about AR gene mobilization. According to the literature, class 1, 2, and 3 integrons are historically associated with antimicrobial-resistance gene spread [44]. It has already been suggested that integrons might be associated with resistance to β-lactams, aminoglycosides, quinolones, sulfonamides, macrolides, and tetracyclines [45,46,47]. 

Overall, the high prevalence of antibiotic resistance genes in MFC samples raises issues about its clinical resistome composition and the threat of the cheese ecosystem as a potential reservoir of resistance genes. In addition, the presence of class 1 and 2 integrons may lead to the risk of AR gene mobilization to human intestinal microbiota and putative pathogens. A larger scale investigation with metagenomic studies would clarify correlations between the bacterial community of MFC and the resistance markers detected in this study.

## Supplementary Materials

The following are available online at http://www.mdpi.com/2073-4425/9/2/106/s1, Table S1: Primers used in this study.
