# Supplementary material for: Antibiotic Resistance Genetic Markers and Integrons in White Soft Cheese: Aspects of Clinical Resistome and Potentiality of Horizontal Gene Transfer
Source: Genes (Basel). 2018 Feb 19;9(2):106. doi: 10.3390/genes9020106 (PMC5852602; doi:10.3390/genes9020106)
Supplement: Supplementary file 1 [file genes-09-00106-s001.docx]

Supplementary Material: Antibiotic Resistance Genetic Markers and Integrons in White Soft Cheese: Aspects of Clinical Resistome and Potentiality of Horizontal Gene Transfer

Ana Caroline L. de Paula, Julliane D. Medeiros, Analice C. de Azevedo, Jéssica M. de Assis Chagas, Vânia L. da Silva and Cláudio G. Diniz *

**Table S1.** Primers used in this study.

| **Target Gene** | **Class of antibiotic/ integron** | **Primer Sequence (5’ – 3’)** | **Amplicon (bp)** | **Reference** |
| --- | --- | --- | --- | --- |
| *bla_CTX-M_* | β-lactam | ATG TGC AGY ACC AGT AAA G  GGT CAC CAG AAG GAG C | 562 | [1] |
| *bla_KPC_* | β -lactam | ATG TCA CTG TAT CGC CGT CT  TTT TCA GAG CCT TAC TGC CC | 892 | [1] |
| *bla_SHV_* | β -lactam | CTT TAC TCG CCT TTA TCG GC  TTA CCG ACC GGC ATC TTT CC | 982 | [1] |
| *bla_TEM_* | β -lactam | GTG CGC GGA ACC CCT ATT  TTA CCA ATG CTT AAT CAG TGA GGC | 968 | [1] |
| *bla_OXA-23_* | β -lactam | GAT CGG ATT GGA GAA CCA GA  ATT TCT GAC CGC ATT TCC AT | 501 | [2] |
| *bla_OXA-51_* | β -lactam | TAA TGC TTT GAT CGG CCT TG  TGG ATT GCA CTT CAT CTT GG | 353 | [2] |
| *bla_OXA-24_* | β -lactam | GGT TAG TTG GCC CCC TTA AA  AGT TGA GCG AAA AGG GGA TT | 246 | [2] |
| *bla_OXA-143_* | β -lactam | TGG CAC TTT CAG CAG TTC CT  TAA TCT TGA GGG GGC CAA CC | 149 | [3] |
| *bla_OXA-58_* | β -lactam | AAG TAT TGG GGC TTG TGC TG  CCC CTC TGC GCT CTA CAT AC | 599 | [2] |
| *blaZ* | β -lactam | ACT TCA ACA CCT GCT GCT TTC  TGA CCA CTT TTA TCA GCA ACC | 173 | [4] |
| *cfxA/cfxA2* | β -lactam | CGT AGT TTT GAG TAT AGC TTT  GAT GTT GCC TAT ATA TGT C | 802 | [5] |
| *ampC* | β -lactam | ATA ACC ACC CAG TCA CGC  CAG TAG CGA GAC TGC GCA | 630 | [6] |
| *bla*SPM-1 | β -lactam | CCT ACA ATC TAA CGG CGA CC  TCG CCG TGT CCA GGT ATA AC | 649 | [7] |
| *cfi*A | β -lactam | TCC ATG CTT TTC CCT GTC GCA GTT AT  GGG CTA TGG CTT TGA AGT GC | 683 | [13] |
| *tet*(A) | Tetracycline | GCT ACA TCC TGC TTG CCT TC  CAT AGA TCG CCG TGA AGA GG | 210 | [9] |
| *tet*(B) | Tetracycline | TTG GTT AGG GGC AAG TTT TG  GTA ATG GGC CAA TAA CAC CG | 659 | [9] |
| *tet*(E) | Tetracycline | AAA CCA CAT CCT CCA TAC GC  AAA TAG GCC ACA ACC GTC AG | 278 | [9] |
| *tet*(K) | Tetracycline | GTA GCG ACA ATA GGT AAT AGT  GTA GTG ACA ATA AAC CTC CTA | 360 | [10] |
| *tet*(L) | Tetracycline | TCG TTA GCG TGC TGT CAT TC  GTA TCC CAC CAA TGT AGC CG | 267 | [9] |
| *tet*(M) | Tetracycline | AGT GGA GCG ATT ACA GAA  CAT ATG TCC TGG GGT GTC TA | 158 | [10] |
| *tet*(O) | Tetracycline | AGC GTC AAA GGG GAA TCA CTA TCC  CGG CGG GGT TGG CAA ATA | 1723 | [11] |
| *tet*(Q) | Tetracycline | TTA TAC TTC CTC CGG CAT CG  ATC GGT TCG AGA ATG TCC AC | 904 | [9] |
| *mrsA* | Methicillin | TCC AAT CAT AGC ACA AAA TC  AAT TCC CTC TAT TTG GTG GT | 163 | [12] |
| *mecA* | Methicillin | GTA GAA ATG ACT GAACGT CCGATA A  CCA ATT CCA CAT TGT TTC GGT CTA A | 310 | [13] |
| *ereA* | Erythromycin | AAC ACC CTG AAC CCA AGG GAC G  CTT CAC ATC CGG ATT CGC TCG A | 420 | [14] |
| *ereB* | Erythromycin | AGA AAT GGA GGT TCA TAC TTA CCA  CAT ATA AAT CAT CAC CAC CAA TGG CA | 546 | [14] |
| *mphA* | Erythromycin | AAC TGT ACG CAC TTG C  GGT ACT CTT CGT TAC C | 837 | [14] |
| *ermA* | MLS* | AAG CGG TAA ACC CCT CTG A  TTC GCA AAT CCC TTC TCA AC | 190 | [10] |
| *ermB* | MLS* | CTA TCT GAT TGT TGA AGA AGG ATG AAA  GTT TAC TCT TGG TTT AGG ATG AAA | 142 | [4] |
| *qnrB* | Quinolone | GAT CGT GAA AGC CAG AAA GG  ATG AGC AAC GAT GCC TGG TA | 476 | [15] |
| *qnr*S | Quinolone | GCA AGT TCA TTG AAC AGG GT  TCT AAA CCG TCG AGT TCG GCG | 428 | [15] |
| *sul*1 | Sulfonamide | ATG GTG ACG GTG TTC GGC ATT CTG A CTA GGC ATG ATC TAA CCC TCG GTC T | 815 | [16] |
| *sul2* | Sulfonamide | CCT GTT TCG TCC GAC ACA GA  GAA GCG CAG CCG CAA TTC AT | 396 | [17] |
| *aacA-aphD* | Aminoglycosides | TAA TCC AAG AGC AAT AAG GGC  GCC ACA CTA TCA TAA CCA CTA | 227 | [10] |
| *Vgb* | MLS^*^ | ACT AAC CAA GAT ACA GACGAGC  TTA TTG CTT GTC AGC CTT CC | 734 | [18] |
| *cep*A | MLS^*^ | TTT CTG CTA TGT CCT GCC C  ATC TTT CAC GAA GAC GGC | 743 | [19] |
| *mexB* | Efflux pumps | GTG TTC GGC TCG CAG TAC TC  AAC CGT CGG GAT TGA CCT TG | 244 | [20] |
| *mexD* | Efflux pumps | CGA GCG CTA TTC GCT GC  GGC AGT TGC ACG TCG A | 165 | [21] |
| *mexF* | Efflux pumps | CGC CTG GTC ACC GAG GAA GAG T  TAG TCC ATG GCT TGC GGG AAG C | 255 | [22] |
| *mexY* | Efflux pumps | CCG CTA CAA CGG CTA TCC CT  AGC GGG ATC GAC CAG CTT TC | 250 | [20] |
| *IntI1* | Class 1 integron | GGT CAA GGA TCT GGA TTT CG  ACA TGC GTG TAA ATC ATC GTC | 436 | [23] |
| *Intl2* | Class 2 integron | CAC GGA TAT GCG ACA AAA AGG  TGTA GCA AAC GAG TGA CGA AAT G | 788 | [23] |
| *Int3* | Class 3 integron | AGT GGG TGG CGA ATG AGT G  TGT TCT TGT ATC GGC AGG TG | 600 | [23] |

* Macrolide, Lincosamide, Streptogramin

References

1. Jones, C.H.; Tuckman, M.; Keeney, D.; Ruzin, A.; Bradford, P.A.; Characterization and sequence analysis of extended-spectrum-β-lactamase-encoding genes from *Escherichia coli, Klebsiella pneumoniae*, and *Proteus mirabilis* isolates collected during tigecycline phase 3 clinical trials. *Antimicrob. Agents Chemother.* **2009**, *53*, 465–475, DOI: 10.1128/AAC.00883–08.
2. Woodford, N.; Ellington, M.J.; Coelho, J.M.; Turton, J.F.; Ward, M.E.; Brown, S.; Amyes, S.G.B.; Livermore, D.M. Multiplex PCR for genes encoding prevalent OXA carbapenemases in *Acinetobacter* spp. *Int. J. Antimicrob. Agents* **2006**, *27*, 351–353.
3. Higgins, P.G.; Poirel, L.; Lehmann, M.; Nordmann, P.; Seifert, H.O. Inclusion of *OXA-143* primers in a multiplex polymerase chain reaction (PCR) for genes encoding prevalent OXA carbapenemases in *Acinetobacter* spp. *Int. J. Antimicrob. Agents* **2010**, *35*, 305–314, DOI: 10.1016/j.ijantimicag.2009.10.014.
4. Martineau, F.; Picard, F.J.; Lansac, N.; Ménard, C.; Roy, P.H.; Ouellette, M.; Bergeron, M.G. Correlation between the resistance genotype determined by multiplex PCR assays and the antibiotic susceptibility patterns of *Staphylococcus aureus* and *Staphylococcus epidermidis*. *Antimicrob. Agents Chemother.* **2000**, *44*, 231–238, DOI: 10.1128/AAC.44.2.231–238.2000.
5. Giraud-Morin, C.; Madinier, I.; Fosse, T. Sequence analysis of cfxA2-like β-lactamases in Prevotella species. *Antimicrob. Agents Chemother.* **2003**, *51*,1293–1296, DOI: 10.1093/jac/dkg221.
6. Odeh, R.; Kelkar, S.; Hujer, A.M.; Bonomo, R.A.; Schreckenberger, P.C.; Quinn, J.P.; Broad resistance due to plasmid-mediated AmpC β-Lactamases in clinical isolates of Escherichia coli. Clin. Infec. Dis. **2002**, *35*, 140–145, DOI: 10.1086/340742.
7. Gales, A.C.; Menezes, L.C.; Silbert, S.; Sader, H.S. Dissemination in distinct Brazilian regions of an epidemic carbapenem-resistant Pseudomonas aeruginosa producing SPM metallo-β-lactamase. J. Antimicrob. Chemother. **2003**, *52*, 699–702, DOI: 10.1093/jac/dkg416.
8. [Sóki](http://jmm.microbiologyresearch.org/search?value1=J%C3%B3zsef+S%C3%B3ki&option1=author&noRedirect=true), J.; [Fodor](http://jmm.microbiologyresearch.org/search?value1=Eleon%C3%B3ra+Fodor&option1=author&noRedirect=true), E.; [Hecht](http://jmm.microbiologyresearch.org/search?value1=David+W.+Hecht&option1=author&noRedirect=true), D.W.; [Edwards](http://jmm.microbiologyresearch.org/search?value1=Richard+Edwards&option1=author&noRedirect=true), R.; [Rotimi](http://jmm.microbiologyresearch.org/search?value1=Vincent+O.+Rotimi&option1=author&noRedirect=true), V.O.; [Kerekes](http://jmm.microbiologyresearch.org/search?value1=Ir%C3%A9n+Kerekes&option1=author&noRedirect=true), I.; [Urbán](http://jmm.microbiologyresearch.org/search?value1=Edit+Urb%C3%A1n&option1=author&noRedirect=true), E.; [Nagy](http://jmm.microbiologyresearch.org/search?value1=Elisabeth+Nagy&option1=author&noRedirect=true), E. Molecular characterization of imipenem-resistant, *cfiA*-positive *Bacteroides fragilis* isolates from the USA, Hungary and Kuwait. *J. Med. Microbiol.* **2004**, *53*, 413–419, DOI: 10.1099/jmm.0.05452–0.
9. Ng, L.K.; Martin, I.; Alfa, M.; Mulvey, M. Multiplex PCR for the detection of tetracycline resistant genes. *Mol. Cell. Probes* **2001**, *15*, 209–215, DOI: 10.1006/mcpr.2001.0363.
10. Strommenger, B.; Kettlitz, C.; Werner, G.; Witte, W. Multiplex PCR assay for simultaneous detection of nine clinically relevant antibiotic resistance genes in *Staphylococcus aureus*. *J. Clin. Microbiol.* **2003**, *41*, 4089–4094, DOI: 10.1128/JCM.41.9.4089–4094.
11. [Trzcinski, K](https://www.ncbi.nlm.nih.gov/pubmed/?term=Trzcinski%20K%5BAuthor%5D&cauthor=true&cauthor_uid=10837427).; [Cooper, B.S](https://www.ncbi.nlm.nih.gov/pubmed/?term=Cooper%20BS%5BAuthor%5D&cauthor=true&cauthor_uid=10837427).; [Hryniewicz, W](https://www.ncbi.nlm.nih.gov/pubmed/?term=Hryniewicz%20W%5BAuthor%5D&cauthor=true&cauthor_uid=10837427).; [Dowson, C.G](https://www.ncbi.nlm.nih.gov/pubmed/?term=Dowson%20CG%5BAuthor%5D&cauthor=true&cauthor_uid=10837427). Expression of resistance to tetracyclines in strains of methicillin-resistant *Staphylococcus aureus*. *J. Antimicrob. Chemother.* **2000**, *45*, 763–70, DOI: 10.1093/jac/45.6.763.
12. Zmantar, T.; Chaieb, K.; Ben Abdallah, F.; Ben Kahla-Nakbi, A.; Ben Hassen, A.; Mahdouani, K.; Bakhrouf, A. Multiplex PCR detection of the antibiotic resistance genes in *Staphylococcus aureus* strains isolated from auricular infections. *Folia Microbiol.* **2008**, *53*, 357–362, DOI: 10.1007/s12223–008–0055–5.
13. Zhang, K.; Sparling, J.; Chow, B.L.; Elsayed, S.; Hussain, Z.; Church, D.L.; Gregson, D.B.; Louie, T.; Conly, J.M. New quadriplex PCR assay for detection of methicillin and mupirocin resistance and simultaneous discrimination of *Staphylococcus aureus* from coagulase-negative *Staphylococci.* *J. Clin. Microbiol.* **2004**, *42*, 4947–4955, DOI: 10.1128/JCM.42.11.4947–4955.2004.
14. Sutcliffe, J.; Grebe, T.; Tait-Kamradt, A.; Wondrack, L. Detection of erythromycin-resistant determinants by PCR. *Antimicrob. Agents Chemother.* **1996**, *40*, 2562–2566.
15. Kim, H.B.; Park, C.H.; Kim, C.J.; Kim, E.-C.; Jacoby, G.A.; Hooper, D.C. Prevalence of plasmid-mediated quinolone resistance determinants over a 9-year period. *Antimicrob. Agents Chemother.* **2009**, *53*, 639–645, DOI: 10.1128/AAC.01051–08.
16. Grape, M.; Sundström, L.; Kronvall, G. Sulphonamide resistance gene sul3 found in Escherichia coli isolates from human sources. J. Antimicrob. Chemother. **2003**, *52*,1022–1024, DOI: 10.1093/jac/dkg473.
17. Hindi, A.K.; Shubbar, E.E.; Addos, S.A. Molecular study on distribution of *sul-1* and *sul-2* genes among *Salmonella enterica* causing enteric fever. *Mag. Al-Kufa Univ. Biol.* **2013**, *5*, 1–9.
18. Lina, G.; Quaglia, A.; Reverdy, M.-E.; Leclercq, R.; Vandenesch, F.; Etienne, J. distribution of genes encoding resistance to macrolides, lincosamides, and streptogramins among *Staphylococci*. *Antimicrob. Agents Chemother.* **1999**, *43*, 1062–1066.
19. Nakano, V.; do Nascimento e Silva, A.; Merino, V.R.C.; Wexler, H.M.; Avila-Campos, M.J. Antimicrobial resistance and prevalence of resistance genes in intestinal *Bacteroidales* strains. *Clinics* **2011**, *66*, 543–547, DOI: 10.1590/S1807–59322011000400004.
20. Yoneda, K.; Chikumi, H.; Murata, T.; Gotoh, N.; Yamamoto, H.; Fujiwara, H.; Nishino, T.; Shimizu, E. Measurement of Pseudomonas aeruginosa multidrug efflux pumps by quantitative real-time polymerase chain reaction. FEMS Microbiol. Lett. **2005**, 243, 125–131, DOI: 10.1016/j.femsle.2004.11.048.
21. Xavier, D.E.; Picão, R.C.; Girardello, R.; Fehlberg, L.C.; Gales, A.C. Efflux pumps expression and its association with porin down-regulation and β-lactamase production among *Pseudomonas aeruginosa* causing bloodstream infections in Brazil. *BMC Microbiol.* **2010**, *10*, 217, DOI: 10.1186/1471–2180–10–217.
22. El Amin, N.; Giske, C.G.; Jalal, S.; Keijser, B.; Kronvall, G.; Wretlind, B. Carbapenem resistance mechanisms in *Pseudomonas aeruginosa*: alterations of porin OprD and efflux proteins do not fully explain resistance patterns observed in clinical isolates. [*APMIS*](https://www.ncbi.nlm.nih.gov/pubmed/15799762) **2005**, *113*, 187–196, DOI: 10.1111/j.1600–0463.2005.apm1130306.x.
23. Machado, E.; Canton, R.; Baquero, F.; Galán, J-C.; Rollán, A.; Peixe, L.; Coque, T.M. Integron content of extended-spectrum-β-lactamase-producing *Escherichia coli* strains over 12 years in a single hospital in Madrid, Spain. *Antimicrob. Agents Chemother.* **2005**, *49*, 1823–1829, DOI: 10.1128/AAC.49.5.1823–1829.2005.
